# Anatomical factors affecting the selection of an operative approach for fibular fractures involving the posterior malleolus

**DOI:** 10.3892/etm.2012.872

**Published:** 2012-12-21

**Authors:** XU WANG, XIN MA, CHAO ZHANG, JIAZHANG HUANG, JIANYUAN JIANG

**Affiliations:** Department of Orthopedics, Huashan Hospital, Fudan University, Shanghai 200040, P.R. China

**Keywords:** ankle joint, fracture, internal fixation

## Abstract

Several operative approaches are available at present for the exposure and fixation of distal fibular fractures combined with posterior malleolus fractures. The present study was designed to study the anatomical characteristics of the distal fibula and to thereby evaluate the advantages and limitations of various operative approaches, as well as their indications for specific conditions. Ten leg specimens from below the knee joint were dissected using posterior, lateral and posterolateral approaches to the fibula. The adjacent vulnerable structures, including nerves, blood vessels, tendons and ligaments, were carefully examined and their distances from the posterior malleolus were recorded. The distance was 7.2±4.1 mm between the sural nerve and the posterior section of the fibula, 79.2±23.5 mm between the lateral malleolus tip and the point where the shape changes in the lower fibula and 66.4±17.4 mm between the lateral malleolus and the jointed tendon of the peroneal and flexor hallux longus muscles. The widest anteroposterior diameter of the distal fibula was 27.3±3.5 mm. Various approaches have certain advantages and limitations when these anatomical factors are taken into account. The choice should be based on the height of the fibular fracture line, the type of posterior malleolus fracture, the effect of the fracture on the stability of the ankle joint and the materials used for internal fixation.

## Introduction

Ankle joint injuries are common in clinical practice (1,2) and lateral malleolar fractures have the highest incidence (3,4). The Danis-Weber classification is a commonly used system for classifying distal fibular fractures. The classification concerns the association of the fracture line with the distal tibiofibular syndesmosis. Type A fractures have a fracture line in the distal fibula which is lower than the syndesmosis level. The fracture line in type B is the same as the syndesmosis level while in type C the syndemosis level is higher (5).

Ankle fractures involving posterior malleolus fractures account for 14–44% of all ankle fractures (6). In 1932, Henderson (8) first defined posterior malleolus fractures and proposed the trimalleolar concept. However, this definition was based on an imaging view from an upper lateral film and was not a truly anatomical concept. Since the average lateral rotation angle of the lateral ankle joint is 27.5°, the majority of the fracture lines are irregular, meaning that the angle between the posterior malleolus fracture line and the lateral axis of the ankle joint detected by a lateral X-ray varies. It is almost impossible to accurately assess the size and orientation of posterior malleolus fracture fragments via X-ray films (8).

In 2006, Haraguchi *et al*(16) classified posterior malleolus fractures into three types based on computed tomography (CT) scans. The type I fractures involved wedge-shaped bone fragments at the posterolateral end of the tibia with transverse fracture lines. The type II fractures included fractures with a fracture line extending from the fibular notch at the lower end of the tibia to the medial malleolus and the type III fractures included solitary or multiple cortical fractures at the posterior lip of the tibia. The ratio of the posterior malleolus fracture fragments to the entire surface area of the tibial distal joint (based on CT scans) was calculated for each type of fracture. Haraguchi *et al*(16) observed that 11.7% of the joint surface was affected in type I fractures, 29.8% in type II fractures and only an extremely small area in type III fractures.

The optimal intervention for posterior malleolus fractures has long been controversial. Certain clinicians believe that additional treatment for posterior malleolus fractures is not necessary since resetting the fracture fragments may be achieved with the reduction of the lateral malleolus fracture due to the mechanical stretch from the ligament behind the distal tibiofibular syndesmosis (10). By contrast, others support the view that incomplete reduction of posterior fractures may alter the contact area and the biomechanics of the tibiotalar joint. These surgeons recommend an incision in the posterior joint capsule which allows the doctor to reset the fracture fragments and fix the screws within a direct view (9). At present, it is commonly accepted that fractures in which >10% (25% in previous criteria) of the joint surface is affected and >2 mm of displacement of the posterior malleolus fracture fragments is evident should be actively treated since fractures involving the posterior malleolus result in osteoarthritis in the ankle joint more frequently than bimalleolar fractures (11,12).

Similarly, dispute remains in terms of the incision selection for fibular fractures accompanied by posterior malleolus fractures. The lateral approach is performed between the superficial peroneal and sural nerves (in front of the peroneus longus and peroneus brevis) and provides good access to the ankle joint, allowing for easy placement of a distal tibiofibular syndesmotic screw (13). The posterolateral incision is performed on the medial side of the posterior edge of the fibula and the point of entry is between the peroneal and flexor hallux longus tendons. Peroneal tendons are usually stretched toward the medial side to expose the fracture fragments at the corner of the posterolateral side of the fibula and the posterolateral side of the posterior malleolus (14). The posterior incision is performed between the Achilles tendon and the distal fibula and the peroneal tendon is stretched toward the lateral side, which protects the sural nerve and the small saphenous vein on the lateral side, while exposing the fibular fracture line and the posterolateral corner of the posterior malleolus between the peroneal and flexor hallucis longus tendons. The present study aimed to quantitatively analyze the anatomical characteristics of the lateral, postero-lateral and posterior approaches to the fibula. Furthermore, the advantages of the operative approaches and the anatomical issues that affect the selection of an operative approach for fibula fractures involving the posterior malleolus were analyzed according to the Danis-Weber classification and the Haraguchi classification for CT scans of posterior malleolus fractures.

## Materials and methods

The present study was conducted in accordance with the Declaration of Helsinki and with approval from the Ethics Committee of Huashan Hospital, Fudan University (Shanghai, China). Written informed consent was obtained from all participants.

Leg specimens from below the knee joint were obtained from 10 fresh cadavers which were provided by the Foot and Ankle Surgeon Training Center of the Chinese Medical Association (Applied Anatomy and Training Center, Huashan Hospital, Fudan University). None of the specimens had any evidence of prior injury or surgery to the leg. All specimens were fresh-frozen and stored at −18°C until they were thawed for use.

**Figure 1.**
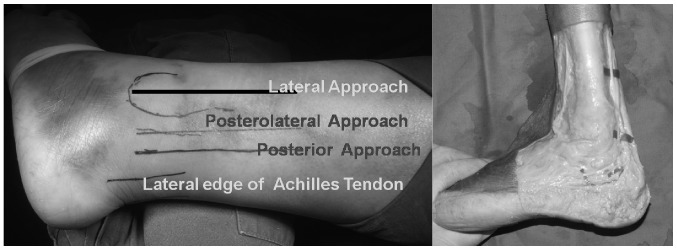
Lateral approach to the fibula located between the sural nerve (labeled) and the superficial peroneal nerve.

Firstly, the skin and superficial fascia were dissected in the posterolateral region of the ankle joint while the sural nerve and superficial peroneal nerve were preserved for further measurements. Then, the posterior, lateral and posterolateral approaches to the fibula were simulated in this region (Fig. 1) and steel plates were placed on the corresponding exposed fibular surface in order to test whether they injured the adjacent structures.

**Figure 2.**
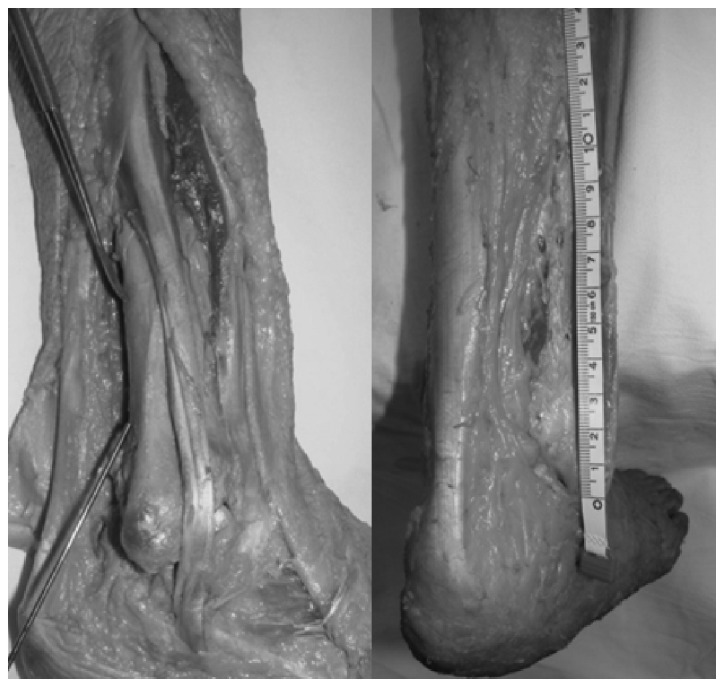
Change of shape at the lower one-third of the fibula and the length of the space between the peroneal tendon and the flexor hallucis longus tendon.

During the procedure, several measurements were taken for each of the specimens, including the distances between the lateral malleolus tip and the location of the transition from a tubular to a triangular shape in the fibula, between the lateral malleolus tip and the muscle belly of the jointed peroneal and flexor hallux longus muscles (Fig. 2) and between the sural nerve and the posterior section of the fibula and the anteroposterior diameter at the widest point of the distal fibula.

## Results

### Distance between the sural nerve and posterior section of the fibula

The distance between the sural nerve and most prominent section of the posterior edge of the lateral malleolus was 7.2±4.1 mm.

### Distance between the lateral malleolus tip and lower fibula

The average distance between the lateral malleolar tip and the point where the shape changes in the lower fibula was 79.2±23.5 mm.

### Distance between the lateral malleolus and the tendons

The distance between the lateral malleolus and jointed tendons of the peroneal and flexor hallux longus muscles was 66.4±17.4 mm.

### Diameter of distal fibular

The anteroposterior diameter at the widest point of the distal fibula was 27.3±3.5 mm (Fig. 5).

### Distance between the fibular tip and superficial peroneal nerve

The distance between the point where the superficial peroneal nerve crossed through the deep fascia and anterior edge of the fibula was 3±3 mm. The height of the point had large variations between individuals. The lowest position occurred at ∼5 cm from the proximal end of the fibula tip.

## Discussion

The lateral incision is the most commonly used approach to access and expose distal fibula in the surgical treatment of malleolar fractures. The measurements taken in the current study showed that the mean distance between the penetration point of the superficial peroneal nerve and anterior edge of the fibula was 3±3 mm, while the horizontal distance between the sural nerve and the most prominent section of the posterior edge of the lateral malleolus was 7.2±4.1 mm. These results demonstrated that the lateral incision was located exactly between these two vulnerable nerves and the exposure area was a relatively safe area in which to place a steel plate (Fig. 1).

**Figure 3.**
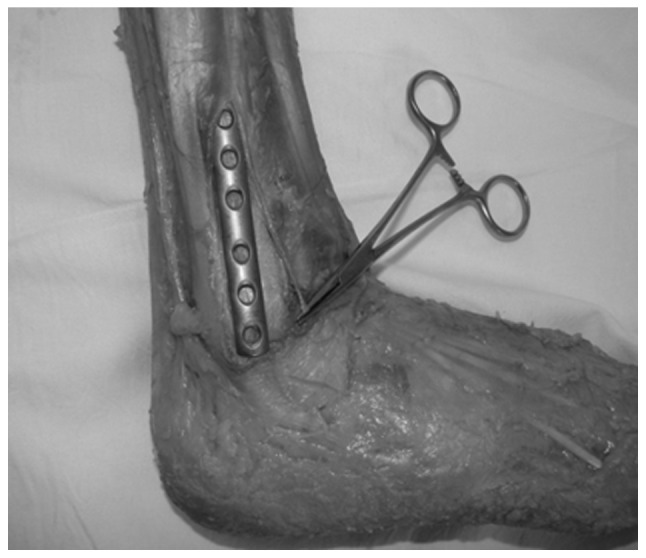
Lateral steel plate may cause stimulation of the superficial peroneal nerve.

However, the height of the penetrating position of the superficial peroneal nerve varied greatly. The lowest position was ∼5 cm proximal to the fibula tip. This result was similar to that of Kim *et al*(15) who also reported a 5-cm distance to where the nerve penetrated the deep fascia. Thus, if a steel plate placed on the lateral side of the fibula is too long, the plate may stimulate the superficial peroneal nerve in individuals with a low penetrating point (Fig. 3).

**Figure 4.**
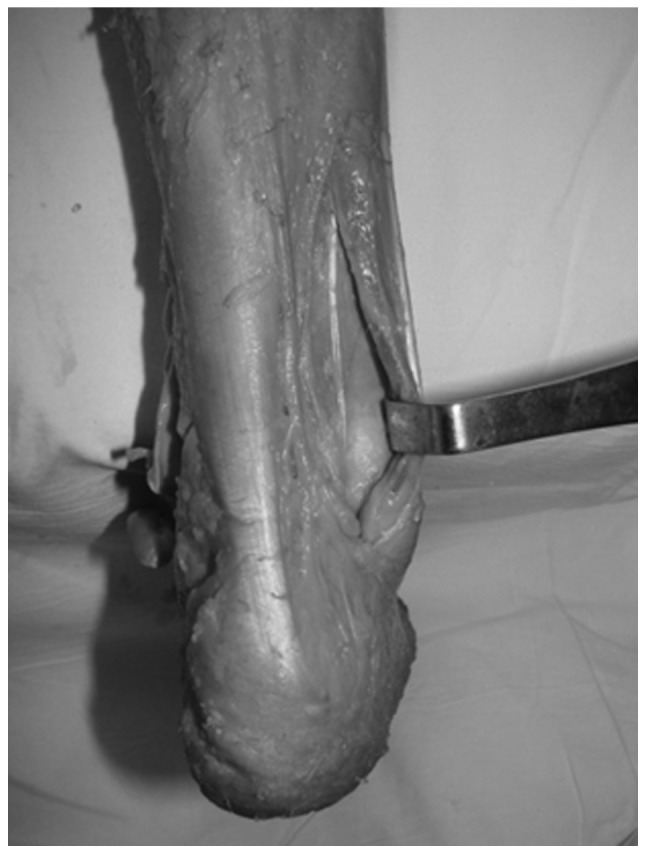
Incision at the posterior fibula.

**Figure 5.**
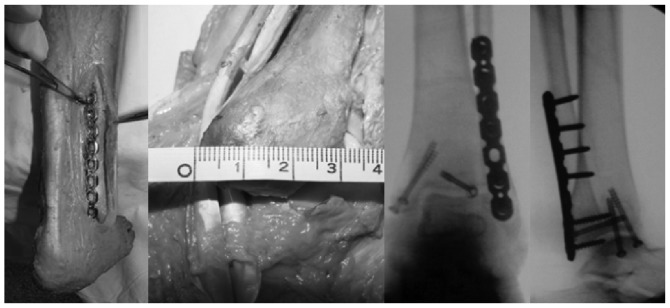
Position of the steel plate and the anteroposterior diameter of the lateral malleolus in the posterior approach to the fibula.

The distal end of the posterolateral incision is adjacent to the sural nerve and the small saphenous vein and the surgeon should be careful in this area (16). The majority of the posterior malleolus may be exposed with the separation of the flexor hallucis longus tendon from the posterior malleolus (Fig. 4), although perforators of the peroneal artery and the accompanying veins were occasionally observed at the distal part of this incision.

The Danis-Weber classification system not only indicates the injuries relative to the distal tibiofibular syndesmosis, but also aids surgeons in selecting the location of the fibular incision (7,17). For type A and B fractures, the steel plate is placed on the lateral side of the fibula using a lateral incision and the distal end is usually fixed with one or two unicortical cancellous bone screws. The lateral placement of the steel plate on the lateral side of the fibula facilitates the screw placement in the tibiofibular syndesmosis and stabilizes the reduced tibiofibular syndesmosis. However, it does not provide a direct visual field for reduction and fixation under the conditions of combined posterior malleolus fractures.

**Figure 6.**
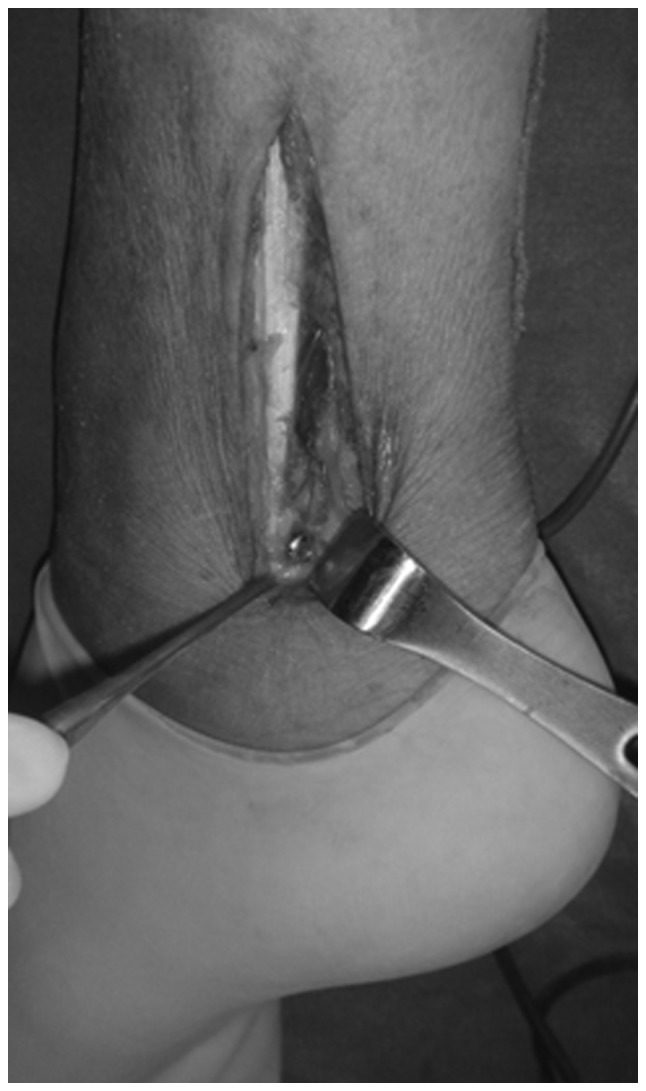
Peroneal tendon irritation caused by the distal screws of the steel plate at the posterior fibula.

The distal fibula and the ligament behind the distal tibiofibular syndesmosis were directly visible for anatomical reduction through a posterior incision and it was possible to place a steel plate at the outer posterior peroneal tendon groove. Since the anteroposterior diameter of the lateral malleolus was 27.3±3.5 mm according to the present measurements, it was possible to place two 25–30-mm cancellous bone screws at the distal fracture line for bicortical fixation. Commonly used large-end screws should be avoided in such conditions since they may cause peroneal tendon irritation (Fig. 6). Posterolateral incisions are not appropriate for type A or B fractures due to the blockage of the peroneal tendon.

For type C fractures, the incision choice should be based on the height of the fracture line. If the fracture line is located below one-third of the distal end of the fibula, we suggest a posterior approach as the preferred choice and a steel plate may be placed under such conditions by properly rotating the lower fibula. This suggestion is mostly based on the present measurement results. The cross-section of the distal fibula diaphysis gradually transforms from a round shape to a triangular shape at a height of 79.2±23.5 mm relative to the tip. Thus, plates placed on either the posterior or the lateral side may encounter a ridge. However, a flat plane may be obtained on the posterior side after the distal fibula is appropriately rotated. If the fracture line is above one-third of the distal end of the fibula, a lateral fibular incision would be more suitable for these patients. This suggestion is mainly due to the observation that the fibers between the peroneal and flexor hallux longus muscles were intertwined with each other at this segment. Therefore, the posterior approach, which requires a separation of the peroneal and flexor hallux longus muscles, may aggravate the injury and cause hemorrhaging in the flexor hallux longus muscle.

Furthermore, in patients with trimalleolar fractures, a posterior approach to the fibula requires the surgeon to change the patient’s position during surgery, increasing the complexity of the procedure.

Since the majority of posterior malleolus fracture fragments are reset with the lateral malleolus using the pulling tension from the ligament behind the distal tibiofibular syndesmosis, clinicians commonly use hollow screws for anteroposterior fixation. However, this is an indirect reset and does not completely confirm the anatomical reduction and stabilization of the fracture. The conventional lateral approach to the fibula has its limitations in the exposure of posterior malleolus fracture fragments as it cannot provide a direct view of them. However, the posterolateral as well as the posterior approach may achieve an improved exposure.

According to the Haraguchi classification system, the posterolateral approach is suitable for type I posterior malleolus fractures due to its complete exposure of the posterolateral corner of the posterior malleolus. For the type II fractures commonly observed in clinical practice, the postero-lateral approach provides limited exposure of the fracture fragments on the medial side. The posterior approach is able to completely expose the posterior malleolus fracture line and provide a direct view of the resetting.

The anatomy of the distal fibula indicates that the selection of an incision is affected by multiple factors for the commonly observed fibular fractures accompanied by posterior malleolus fractures. Although the fracture site, the fracture shape and the internal fixation must be considered, so too must the surgical techniques that the surgeon is familiar with and able to perform. Moreover, other conditions associated with ankle joint stabilization should be considered, such as whether to explore and repair the medial triangular ligament and whether to use a distal tibiofibular syndesmosis screw. The goal is to restore the stability of the ankle joint and provide good ankle joint function. The clinical application of new types of internal fixation material is also likely to promote progress in the treatment of ankle fractures.
